# Integration of Metabolomics and Transcriptomics Reveals the Therapeutic Mechanism Underlying Paeoniflorin for the Treatment of Allergic Asthma

**DOI:** 10.3389/fphar.2018.01531

**Published:** 2019-01-18

**Authors:** Qiyang Shou, Lu Jin, Jiali Lang, Qiyuan Shan, Zhunan Ni, Changpei Cheng, Qinglin Li, Huiying Fu, Gang Cao

**Affiliations:** ^1^Affiliated Secondary Hospital, Zhejiang Chinese Medical University, Hangzhou, China; ^2^School of Pharmacy, Zhejiang Chinese Medical University, Hangzhou, China; ^3^Institute of Comparative Medicine, Zhejiang Chinese Medical University, Hangzhou, China; ^4^Zhejiang Provincial Center for Disease Control and Prevention, Hangzhou, China; ^5^The Graduate School, Tianjin University of Traditional Chinese Medicine, Tianjin, China; ^6^Zhejiang Cancer Hospital, Hangzhou, China

**Keywords:** paeoniflorin, allergic asthma, metabolomics, transcriptomics, fatty acid metabolism

## Abstract

**Objectives:** Asthma is a chronic airway inflammatory disease, which is characterized by airway remodeling, hyperreactivity and shortness of breath. Paeoniflorin is one of the major active ingredients in Chinese peony, which exerts anti-inflammatory and immune-regulatory effects in multiple diseases. However, it remains unclear whether paeoniflorin treatment can suppress allergic asthma.

**Methods:** In this study, we evaluated the effect of paeoniflorin on lung function and airway inflammation in asthmatic mice. These asthmatic Balb/c mice were first sensitized and constructed through ovalbumin (OVA) motivation. Subsequently, we determined the mechanism of action of paeoniflorin in treating allergic asthma through integrated transcriptomic and metabolomic data sets.

**Results:** Our results demonstrated that many genes and metabolites were regulated in the paeoniflorin-treated mice. Moreover, the potential target proteins of paeoniflorin played important roles in fatty acid metabolism, inflammatory response, oxidative stress and local adhesion.

**Conclusion:** Paeoniflorin has a beneficial effect on asthma, which may be achieved through regulating fatty acid metabolism, inflammatory response and the adhesion pathway at system level.

## Introduction

Allergic asthma is a chronic inflammatory airway disease characterized by airway remodeling, hyperreactivity and short of breath (Morjaria et al., 2018). Currently, three main prescriptions are used to treat asthma patients, which include inhaled short-acting and long-acting β2 agonists, inhaled and oral corticosteroids, and leukotrienes agonists (Gibeon and Menzies-Gow, 2013; Telia et al., 2018). However, these treatments are not effective for all patients (Tan et al., 2016). What’s worse, remarkable, reproducible and undesirable side effects of systemic corticosteroids can also be seen in treatment (Luo et al., 2017; Mao et al., 2017). Therefore, searching for a new antiasthmatic agent is a furious research field.

Over the past thousands of years, Chinese herbal medicine has been used to treat allergic disease, and the effect of herbal medicine has attracted increasing attention (Li et al., 2004; Lu et al., 2016; Wang et al., 2016; Yu et al., 2017; Liu et al., 2018; Yan et al., 2018). Paeoniflorin is one of the major active ingredients in Chinese peony, which is also called Paeonia lactiflora. Paeoniflorin is shown to have anti-inflammatory and immune-regulatory effects (Zhang et al., 2017; Chen et al., 2018). The latest study indicates that paeoniflorin can improve the IgE-induced allergic reaction and scratching behavior (Lee et al., 2008). However, the effectiveness of paeoniflorin for treatment of allergic asthma is rarely known. Therefore, the current study was thereby carried out, aiming to obtain *in vivo* data to suggest that paeoniflorin could improve lung function and mitigate inflammation in allergic asthma mice, and to determine the mechanism of action of paeoniflorin on allergic asthma treatment through integrating the transcriptomic and metabolomic data sets.

## Materials and Methods

### Reagents and Animals

Paeoniflorin was purchased from Mansite Biological Technology Co., Ltd., (Chengdu, China), which had the molecular formula of C_23_H_28_O_11_ and the molecular weight of 480.45. The purity for each standard compound was greater than 98% by HPLC analysis. 4- to 6-week-old female BALB/c mice (18–22 g in weight) were obtained from Shanghai SLAC Laboratory Animal Co., Ltd. This study was conducted in strict accordance with the recommendations of the Guide for the Care and Use of Laboratory Animals of the National Institutes of Health. The experimental animal protocols were approved by the Board of the Animal Study of Zhejiang Chinese Medical University. The animals were raised under the temperature- and humidity-controlled environment without specific pathogens and were allowed free access to food and water. The mice were raised for 7 days prior to experiment to adapt to the environment.

### Establishment and Grouping of Allergic Asthma Model in Mice and Drug Administration

A total of 21 male BALB/c mice were sensitized with 0.2 mg/ml of OVA prepared by 0.4% aluminum hydroxide gel. Seven points, that is: two points on the vola pedis of hind feet, two point on groins, two points on the back, and one point on the abdominal cavity, were observed on each mouse. A total of 0.5 ml solution of 100 μg of OVA and 20 mg of aluminum hydroxide gel was injected (0.2 ml for the abdominal cavity and 0.05 ml for all other points). On the 23rd day, mice were subjected to continuous atomization of 10 mg/ml OVA for 30 min once a day for 7 days. All mice were divided into two groups, that is, model control and paeoniflorin groups. Normal BALB/c mice were selected as the normal control group. The Paeoniflorin group were supplied with 50 mg/kg paeoniflorin, and each index was observed after continuous drug supply for 1 week.

### Lung Function Testing When the Mice Were at Wake Status

After OVA pulverization for 7 days and the final 24 h OVA stimulation of mice, we tested the airway hyper-responsiveness of mice that were awake and non-invasive by systemic plethysmography. We prepared 200 mg/ml of the mother solution of acetylcholine (ACh) with PBS. We then diluted ACh to 25 and 50 mg/ml, placed mice onto the plethysmograph room for 5 min, and recorded the baseline reading for 3 min. A total of 1 ml of PBS were pulverized through a nebulizer. Mice were atomized and inhaled for 2 min, and lung function was observed for 6 min. We removed the remaining PBS in the nebulizer added 1 ml of 25 and 50 mg/ml ACH into the nebulizer and atomized for 2 min. After a 6 min monitoring period, we repeated pulverization and subjected mice to methacholine inhalation. We took out the mice, cleaned the device, and performed the test on the second group of mice. We analyzed the changes in airway resistance (Penh) of mice in each group after ACh pulverization with different concentrations, and we also analyzed the pulmonary function data using lung function analysis software.

### Bronchoalveolar Lavage and Leukocyte Counting and Sorting

After blood was obtained from the mice, they were injected with bronchoalveolar lavage fluid (BLAF) and anesthetized. Atrachea cannula was used, and the bronchi of the right lung was ligated to prevent lavage fluid from entering (the right lung was used for pathological and cell factor determination). A total of 3 ml of heparin-containing Hanks solution was injected into the airway three times via tracheal intubation and 1 ml of the same solution was injected once. After being washed back and forth thrice at a time, the washing liquid was collected in the test tube. For leukocyte and differential counting, perfusate was diluted by 1% glacial acetic acid proportionally, and the amount of leukocytes were counted under the microscope using a counting plate. Perfusate was mounted on the slide and dyed with Wright–Giemsa dye after it was dried. Differential counting was performed under a high-powered lens. We then calculated the oxyphile–leukocyte ratio.

### Chemotactic Factor and Inflammatory Factor Level in Multiterm Serum and Lung Tissue of Mice Were Tested by Liquid-Phase Chip Technology

Mice were anesthetized, heart blood was collected and centrifuged for 10 min at 3000 rpm, and serum was obtained. We acquired lung tissue to prepare 10% lung tissue homogenate, and we obtained lung tissue supernatant after centrifugation. We tested the presence of eotaxin, macrophage inflammatory protein 1α (MIP-1α), interleukin 4 (IL-4), and interleukin 17 (IL-17) via Bio-Plex Pro cell factor. We tested each index level in the serum and lung tissue supernatant by Luminex liquid-phase chip technology, Bio-Plex 2000 system, and high-throughput analysis platform (Bio-Rad company), according to the manufacturer’s instruction.

### Metabolomics Data Collection and Analysis

Lung tissues were extracted using the two-step method. Each left lung tissue sample was homogenized at a rotation speed of 30,000 rpm in 2000 μL of primary freezing extraction solvent (mixture of chloroform, ethanol and water at the ratio of 1:2:1, v/v/v) using the homogenizer (IKA, Staufen, Germany), followed by 5 min of vortex and 15 min of centrifugation at 6000 rpm and 4°C. 1500 μL of supernatant was absorbed into the fresh tube, and the sediment was re-homogenized using the ice cold ethanol (2000 μL). 1500 μL centrifuged supernatant was transferred into the above-mentioned tube for drying, and the dry metabolite extractive was dissolved in 300 μL of acetonitrile-water (1:1, v/v). Later, 2 μL aliquot injection was used for LC/MS analysis. Lung tissue samples were analyzed on the Waters Acquity TM Ultra Performance LC system (Waters Corporation, Milford, MA, United States), equipped with a BEH C_18_ column (100 mm × 2.1 mm, 1.7 μm). The temperature of the automatic sampler was maintained at 4°C, and the column compartment temperature was set at 40°C. The mobile phase was constituted by solvents A (2 mM ammonium acetate supplemented in 95% H_2_O/5% acetonitrile + 0.1% acetic acid) and B (2 mM ammonium acetate supplemented in 95% acetonitrile/5% H_2_O + 0.1% acetic acid). The linear gradient program of lung tissue samples was shown as follows: 0–0.5 min, 1% B; 0.5–2 min, 10% B; 2–5 min, 10–60% B; 5–9 min, 60–90% B; 9–12 min, 90–100% B; 12–16 min, washed with 100% B for 16–19 min, and balanced with 1% B at the rate of 0.45 mL/min.

Moreover, mass spectrometry (MS) was conducted using the electrospray ionization (ESI) source under the cation and anion patterns of the Waters SYNAPT G_2_ HDMS (Waters Corp., Manchester,United Kingdom), respectively. The parameter setting was the same as previously mentioned. The capillary voltage was 3.0 KV (ES+) and 3.2 KV (ES-); the sample cone voltage was 40 V; the dry nitrogen was used, and the de-solvent gas velocity and temperature were 800 L/h and 400°C, respectively, the cone gas rate was 50L/h; the source temperature was 100°C; the scanning time and inter-scanning delay were 0.15 and 0.02 s, respectively. Among all analyses at the concentration of 0.5 μg/mL and the flow rate of 5 μL/min, leucine enkephalin was selected as the locking mass (m/z 556.2771 for cation pattern and m/z 554.2615 for anion pattern). The centroid data were collected, and the mass range was m/z 100 to m/z 1500.

### Transcript Profile Analysis

Transcript analysis was performed by RNA-seq as previously described (Ren et al., 2012). Total RNA was isolated and purified using TRIzol reagent (Invitrogen, Carlsbad, CA, United States) following the manufacturer’s procedure. The RNA amount and purity of each sample was quantified using NanoDrop ND-1000 (NanoDrop, Wilmington, DE, United States). The RNA integrity was assessed by Agilent2100 with RIN number > 7.0. The clean reads of RNA nucleotide sequences were generated using TopHat software and miRNA data were aligned in a mirBase database.

### Gene Set Enrichment, Network and Pathway Analyses

The molecular function and biological process enrichment of the transcript were analyzed using GlueGo (Bindea et al., 2009). The molecular pathway enrichment of candidate genes were analyzed through GSEA (Suarez-Farinas et al., 2010). If a *p*-value was ≤ 0.05, the term was deemed as statistically significant. Moreover, Metscape was also used to analyze the integrated pathways of genes and metabonomic data (Gao et al., 2010).

### Statistical Analysis

Data were derived from three independent experiments and expressed as mean ± standard deviation. Statistical analysis was carried out using student’s paired *t*-test and one-way analysis of variance (ANOVA), in order to obtain the differences between the treatment groups. All analyses were performed using the SPSS 16.0 statistical software. A difference of *P* < 0.05 was deemed as statistically significant.

## Results

### Paeoniflorin Reduce Inflammation and Airway Hyperresponsiveness in Allergic Asthma Mice

**FIGURE 1:**
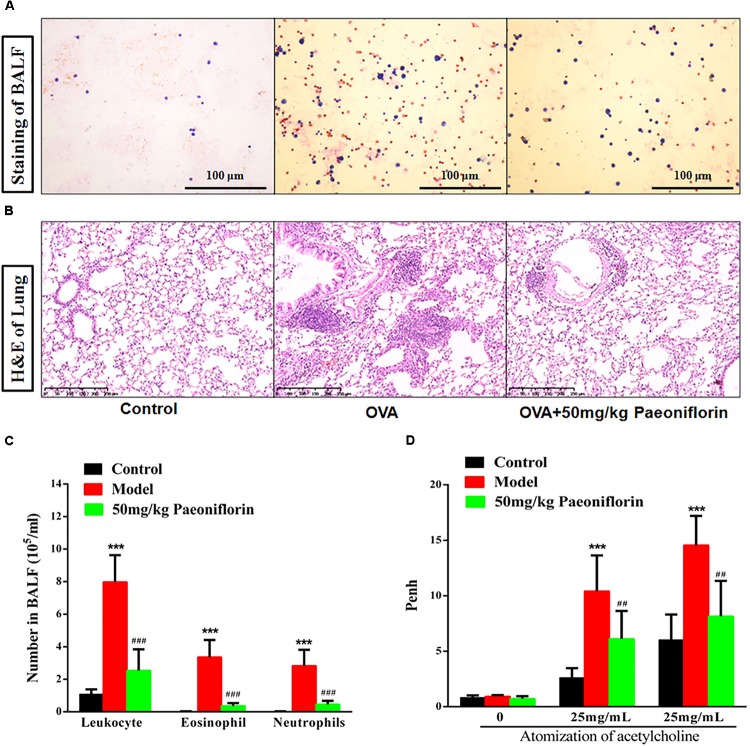
Paeoniflorin reduce inflammation and airway hyperresponsiveness in allergic asthma mice. The staining of BALF **(A)**, lung tissue pathological changes and inflammation inflammatory infiltration **(B)**, Leukocytes, EOS, and neutrophils level in BALF **(C)**, and Penh level **(D)**. The data were expressed as the mean ± SD. ^∗^Means control group vs model group (^∗∗∗^*p* < 0.001); ^#^Means model group vs paeoniflorin group (^##^*p* < 0.01 and ^###^*p* < 0.001).

After the last OVA of the small mouse is activated for 24 h, the right lung is ligated, and the left lung is subjected to PBS lavage. BALF is collected to calculate the number of leukocytes. Leukocyte smear is stained with the Wright–Giemsa compound to observe the leukocyte categories under a microscope. Experimental results show that the numbers of leukocytes, eosinophils (EOS), and neutrophils in the BALF of the model group significantly increased compared with those of the mice in the control group, and the EOS proportion is above 40% (*P* < 0.001). These results indicate that the mouse asthma model induced by OVA is successful. Leukocytes, EOS, and neutrophils can be significantly inhibited in BALF after the mice are given 50 mg/kg paeoniflorin through gavage (*P* < 0.001) (Figures 1A,C).

For the normal mice, the tracheal epithelium and the inner tracheal wall are complete, and no inflammatory cells have infiltrated. For the model mouse, the tracheal epithelium is congested and swollen. Large quantities of inflammatory cells infiltrate around the bronchus. Goblet cells and glands become hyperplastic. Subepithelial collagen and extracellular matrix are deposited. After the mice are given 50 mg/kg paeoniflorin through gavage, pulmonary inflammation in the mice can be significantly inhibited, the number of infiltrated inflammatory cells significantly decreases, and the hyperplasia of the goblet cells and glands is diminished (Figure 1B).

When the model mouse is activated by OVA, the following conditions occur: dysphoria, tachypnea, reduced activities and intake or motionless lying, piloerection, dull fur and even hair loss, reaction retardation, and other asthma symptoms. At the end of the activation process, the mice in the model group exhibit tachypnea, nutation respiratory, and abdominal spasm. After the mice are given 50 mg/kg paeoniflorin through gavage, tachypnea, respiratory nutation, abdominal spasm, and other asthma symptoms can be inhibited. After the last OVA of the small mouse is activated for 24 h, the airway hyperreactivity of the clear mouse is non-invasively measured through whole-body plethysmography to analyze changes in the Penh of each group, after different concentrations of acetylcholine (ACh) are atomized. The Penh of the asthma mouse greatly increases compared with that of the normal control group after activation by different concentrations of ACh (*P* < 0.001). The Penh significantly decreases after the mouse is given paeoniflorin (*P* < 0.001) (Figure 1D).

### Paeoniflorin Reduces Chemokines and Cytokines in Allergic Asthma Mice

**FIGURE 2:**
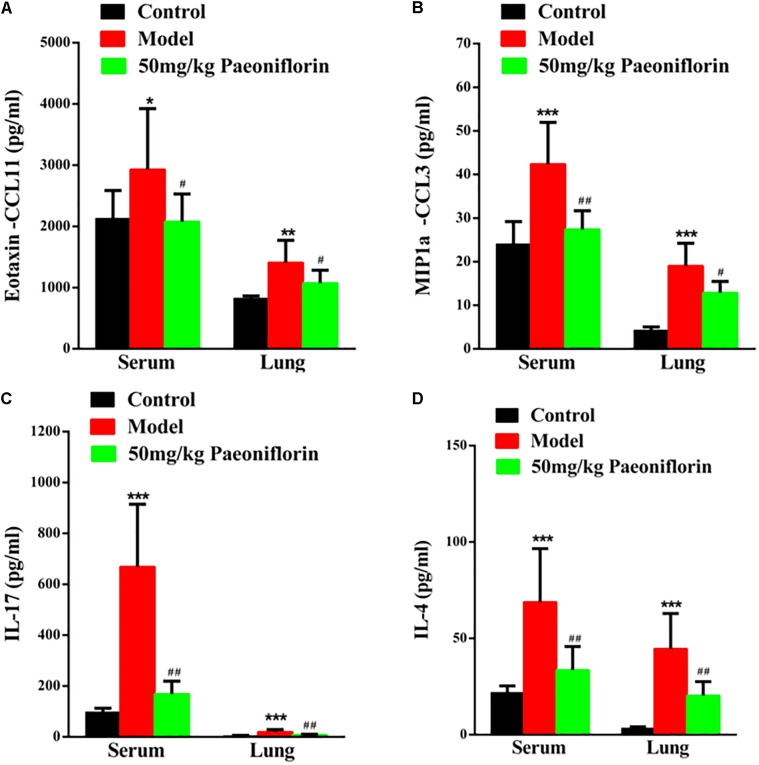
Paeoniflorin reduces chemokines and cytokines in allergic asthma mice. The level of exotaxin in serumand lung tissue **(A)**, the level of MIP-1α in serum and lung tissue **(B)**, the level of IL-17 in serum and lung tissue **(C)**, and the level of IL-4 in serumand lung tissue **(D)**. The data were expressed as the mean ± SD. ^∗^Means control group vs model group (^∗^*p* < 0.05, ^∗∗^*p* < 0.01, and ^∗∗∗^*p* < 0.001); ^#^Means model group vs paeoniflorin group (^#^*p* < 0.05, ^##^*p* < 0.01, and ^###^*p* < 0.001).

Asthma severity is associated with EOS airway inflammation. The EOS chemokine belongs to C–C chemokines (CCL11), which can show chemotactic properties of high selectivity to EOS. In allergic inflammatory responses, exotaxin can specifically collect and activate EOS and achieve EOS chemotaxis. After the mouse is activated through OCA sensitization, exotaxin in the serum and the lung tissue significantly increases (*P* < 0.05, *P* < 0.01). After the mice are given paeoniflorin, exotaxin in the serum and the lung tissue can be significantly inhibited (*P* < 0.05) (Figure 2A).

Macrophage inflammatory protein-1alpha (MIP-1α) plays an important role in asthma airway inflammation, through the specific chemotaxis and activation of all inflammatory cells. MIP-1α belongs to the CC chemokine family, that is, the β chemokine family (CCL3). The encoding gene is located in chromosome No. 11 of mice and chromosome No. 17 of humans. MIP-1α participates in the asthma pathogenic process in the form of a cyclic effect. In particular, when an allergen enters the body, various effector cells, such as macrophages, lymphocytes, and EOS, accumulate and become activated. When the activated cells reach the respiratory tract, they increase in number and release MIP-1α and other chemokines. MIP-1α and other chemokines then participate in the activation and chemotaxis of T cells and EOS. In this experiment, after the mouse is activated by OCA sensitization, the amount of MIP-1α in the serum and the lung tissue significantly increases (*P* < 0.001). After the mice are given paeoniflorin, the increase in the amount of MIP-1α in the serum and the lung tissue can be significantly inhibited (*P* < 0.05, *P* < 0.01) (Figure 2B).

Th 17 plays an important role in the immune responses initiated by autoimmune diseases and many bacteria and fungi, especially mucosal immunity. Therefore, this cell is regarded as a “pro-inflammatory” immune cell. Asthma is a heterogeneous chronic airway inflammatory disease involving many cells. Th 17 and other related cell factors are crucial in asthma pathogenesis. IL-17 is the key pro-inflammatory cytokine of the Th 17 cell, which can promote neutrophil inflammation and assist airway remodeling related to asthma. In this experiment, after the mouse is activated by OCA sensitization, the IL-17 levels in the serum and the lung tissue significantly increase (*P* < 0.001). After the mice are given paeoniflorin, the increase in IL-17 levels in the serum and the lung tissue can be significantly inhibited (*P* < 0.01). After the mouse is activated by OCA sensitization, the amount of IL-4 in the serum and the lung tissue significantly increases (*P* < 0.01) (Figure 2C).

After the mice are given paeoniflorin, the increase in the amount of IL-4 in the serum and the lung tissue of the drug treatment group can be significantly inhibited compared with those of the model control group (*P* < 0.05, *P* < 0.01) (Figure 2D).

### Metabonomic Analysis Results of Paeoniflorin-Treated OVA-Induced Allergic Asthma Mice

To demonstrate the system-wide mechanism of the long-term effect of paeoniflorin, we had detected the metabolomic analysis of lung tissues. Upon metabolomic analysis, 37 metabolites in control, model and paeoniflorin groups had maintained a marked trend of “low-high-low” or “high-low-high,” such as valine, tyrosine, 9,12,15-Octadecatrienoic acid, glutamine, Leukotriene B4, and glutamic acid (Supplementary Figure S1 and Supplementary Tables S1, S2).

**FIGURE 3:**
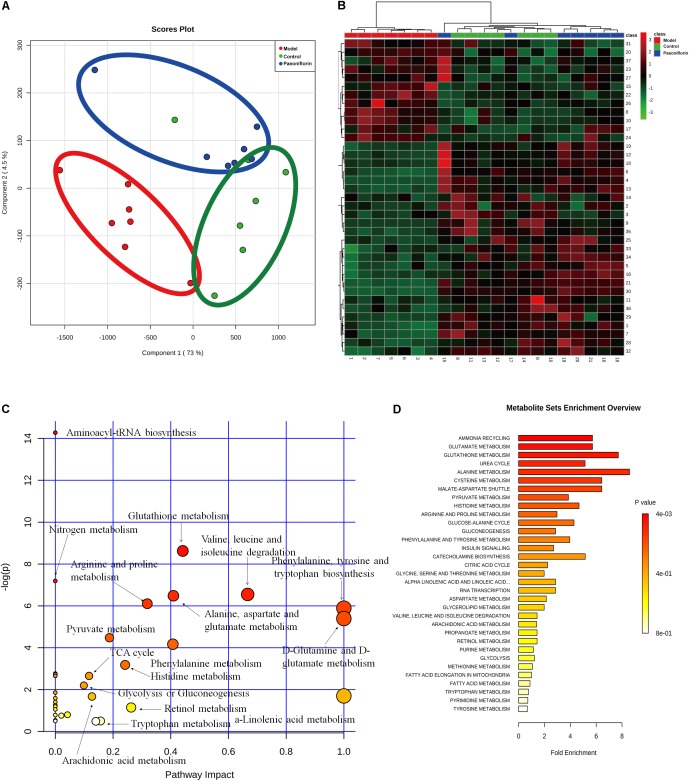
Metabonomic analysis results of paeoniflorin-treated OVA-induced allergic asthma mice. Score plots from the PLS-DA model **(A)** and heatmap **(B)** classifying the control group (Green), model group (Red), paeoniflorin group (Blue) with mice lung samples by UPLC-Q-TOF/MS. Metabolites set enrichment results was shown in **C** and **D**.

To further excavate the underlying meaning of the changing metabolic product, the metabolic pathways were analyzed using MetaboAnalyst (Figures 3A,B). We discovered many markedly changed pathways in the model and paeoniflorin groups at the metabolomic level, including ammonia recycling, glutamate metabolism, urea cycle, cysteine metabolism, alanine metabolism and fatty acid metabolism (Figures 3C,D and Supplementary Table S3).

### Transcriptomics Analysis Results of Paeoniflorin-Treated OVA-Induced Allergic Asthma Mice

Using the microarray-based RNA expression analysis, we had detected about 41000 expressed genes in lung tissues. There were 748 and 419 differentially expressed genes (DEGs) in OVA-induced allergic asthma model (relative to the control) and paeoniflorin treatment (relative to the model) groups.

**FIGURE 4:**
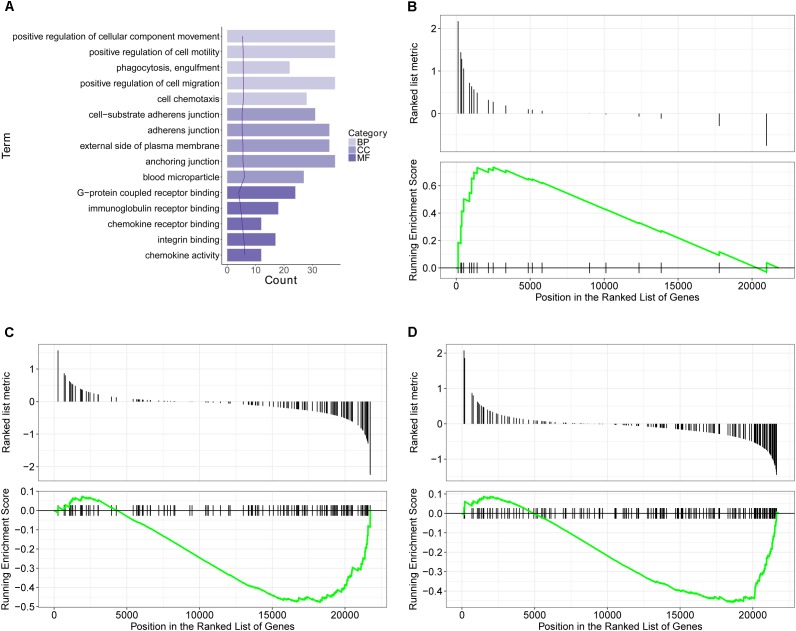
Transcriptomics analysis of OVA on normal mouse. **(A)** GO enrichment analysis of DEGs between Model and Control in BP (Biological process), MF (Molecular function) and Cellular Component (CC). **(B–D)** GSEA analysis of DEGs between Model and Control: **(B)** mmu05310: Asthma; **(C)** mmu04371: Apelin signaling pathway; **(D)** mmu04022: cGMP-PKG signaling pathway.

**FIGURE 5:**
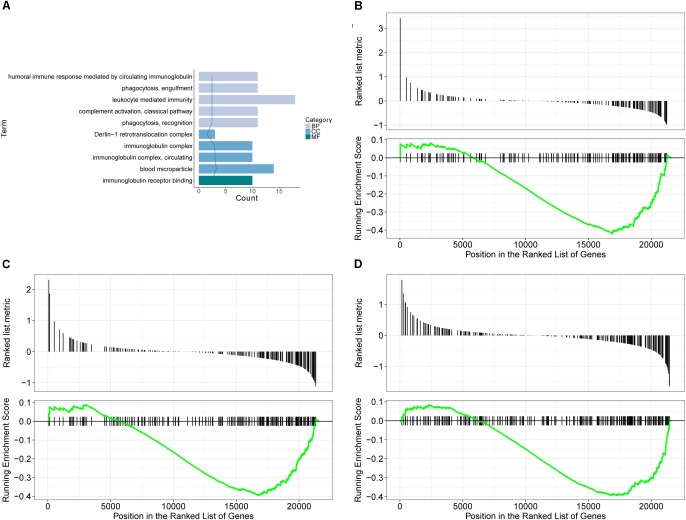
Transcriptomics analysis of paeoniflorin on allergic asthma model mouse. **(A)** GO enrichment analysis of DEGs between Model and Paeoniflorin in BP (Biological process), MF (Molecular function) and Cellular Component (CC). **(B–D)** GSEA analysis of DEGs between Model and Control: **(B)** mmu05152: Tuberculosis; **(C)** mmu04510: Focal adhesion; **(D)** mmu04060: Cytokine-cytokine receptor interaction.

GO database is a widely used database for annotating sequences, genes, and gene products. To better understand the molecular function of genes involved in the response of allergic asthma to paeoniflorin treatment, all the DEGs in both groups of comparison were mapped to terms in the GO database. As shown in Figure 4A, we found that transcripts regulated in the lung tissues of these model mice could be mapped to biological processes (BP) for regulating cell movement ability, cell chemotaxis, cell migration phagocytosis; cell components (CC) for adheren junctions, plasma membrane and blood microparticle; and molecular functions (MF) for receptor binding and chemokine activity. Similarly, as shown in Figure 5A, the transcripts that were only regulated in the paeoniflorin-treated mice could be mapped to BP for phagocytosis, immunity and leukocytes; CC for immunoglobulin complex and Derlin-1 retrotranslocation; MF for immunoglobulin receptor binding.

For further characterization of the DEGs, we performed pathway enrichment analysis with GSEA. As shown in Figures 4B–D, we found that transcripts regulated in the lung tissues of these model mice could be mapped to signaling pathways, such as the Asthma pathway, Apelin signaling pathway, and cGMP-PKG signaling pathway. Nonetheless, the transcripts that were only regulated in the paeoniflorin-treated mice could be mapped to Tuberculosis, Adhesion junctions, and the Cytokine-cytokine receptor interaction pathway, respectively, (Figures 5B–D).

**FIGURE 6:**
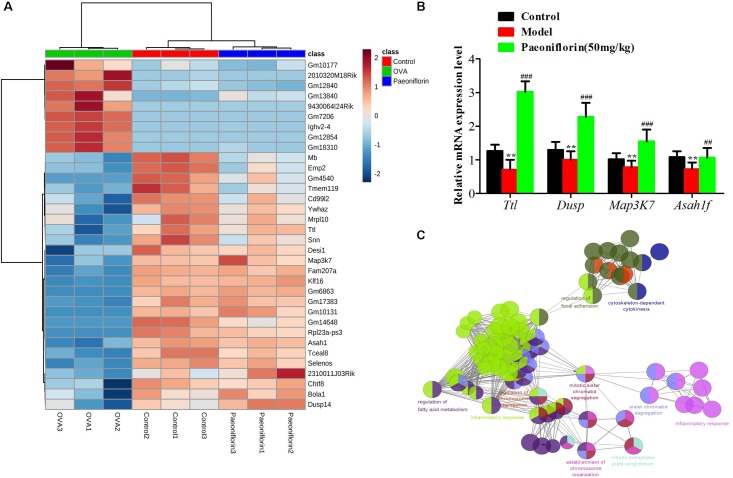
The enrichment analysis of candidate genes of paeoniflorin on allergic asthma model mouse. **(A)** Heatmap classifying the candidate genes of paeoniflorin on allergic asthma model mouse. **(B)** mRNA expression level of *Ttl*, *Dusp*, *Map3k7* and *Asah1f* was detected by real-time PCR assay. **(C)** The genes were mainly related to such biological functions: fatty acid metabolism, inflammation response, cytokinesis, chromosome and adhesion plaque. The data were expressed as the mean ± SD. ^∗^Means control group vs model group (^∗^*p* < 0.05, ^∗∗^*p* < 0.01, and ^∗∗∗^*p* < 0.001); ^#^Means model group vs paeoniflorin group (^#^*p* < 0.05, ^##^*p* < 0.01, and ^###^*p* < 0.001).

Similarly after the metabolomic analysis, we discovered a series of 34 genes in the control, model and paeoniflorin groups that had maintained a distinct trend of “low-high-low” or “high-low-high” (*p* < 0.05 and | log2 FC | > 0.585, Figure 6A). Quantitative real-time PCR (qRT-PCR) analysis partly confirmed these results (Figure 6B). To further illustrate the potential roles of these 34 genes and study the relationship between the functional groups in the biological network and their potential scientific annotations, we used ClueGO, a Cytoscape plugin that visualizes non-redundant biological terms for large clusters of genes in a functionally grouped network. The most vital term in the group is labeled, Functionally related groups partially overlap. Representative enriched pathway *(P* < 0.05) interactions among these 34 genes. As shown in Figure 6C, we discovered that these genes were mainly related to such biological functions: fatty acid metabolism, inflammation response, cytokinesis, chromosome and adhesion plaque.

### Integrated Analysis the Mechanism of Paeoniflorin-Treated OVA-Induced Allergic Asthma Mice From Metabonomics and Transcriptomics Data

**FIGURE 7:**
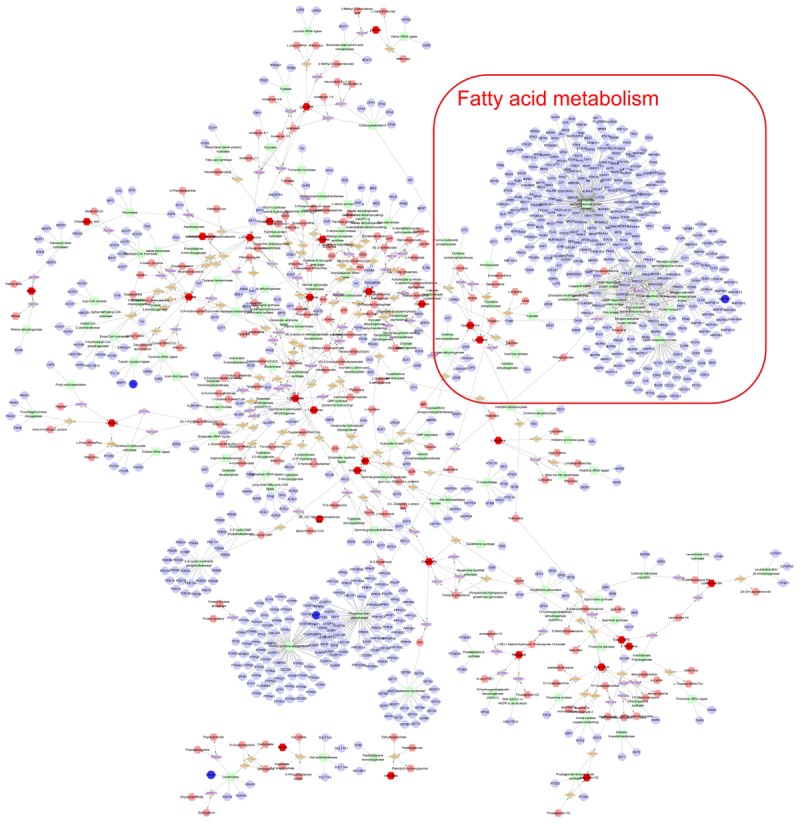
Integrated analysis the mechanism of paeoniflorin-treated OVA-induced allergic asthma mouse from metabonomics and transcriptomics data. Compound reaction networks of the metabolites, genes were visualized using Metscape: metabolites (hexagons), genes (circles), metabolic enzymes (squares) and chemical reactions (grey rhombus) are presented as nodes and relationship are presented as edges. Inputted genes are shown in blue, inputted metabolites are shown in red. The metabolite-gene associated network was mainly related to fatty acid metabolism.

Finally, the Metscape software was used to examine the potential relationship between gene and metabolite measurement. The gene-metabolite network was generated based on the transcriptomic and metabolomic data of mice in the model group and paeoniflorin-treated group. As shown in Figure 7, the metabolite-gene associated network was mainly related to fatty acid metabolism. Such discovery suggested that fatty acid metabolism might be the key biological process related to the development and medical intervention of allergic asthma. In conclusion, we had verified that paeoniflorin could provide protective and therapeutic benefits for allergic asthma through regulating multiple biological functions, such as fatty acid metabolism, inflammatory response and the adhesion pathway.

## Discussion

OVA exposure remains the common method to mimic asthma in mice (Fang et al., 2005; Serra et al., 2018). In this study, the OVA-exposed mice displayed remarkably enhanced bronchial reactivity to methacholine, compared to the control group. Moreover, pathological lung tissue images indicate that inflammatory cells have notably infiltrated into the connective tissues around the bronchiole and blood vessels in the model group. The inflammation score between normal mice and OVA-exposed mice demonstrated a statistically significant difference. These findings verify successful construction of the allergic asthma mouse model.

Lung function data suggest that, compared with OVA-exposed mice, paeoniflorin treatment at the dose of 50 mg/kg can markedly reduce the bronchial reactivity to methacholine, indicating that paeoniflorin treatment can protect the lung function in allergic asthma mice. In our study, several mice have mild diarrhea after paeoniflorin treatment, but no distinct side effect is observed. Furthermore, a clinical experiment indicates that the major adverse effect of paeoniflorin is gastrointestinal disturbance (He and Dai, 2011), which is dominated by mild diarrhea.

Inflammation has long been considered as the major feature of asthma that leads to AHR and airway obstruction, excessive production of mucus and airway wall remodeling (Rosi et al., 2006). Neutrophils have been regarded as the key inflammatory cells in allergic asthma (Radermecker et al., 2018). Paeoniflorin administration at a dose of 50 mg/kg can partly improve the lung histological and inflammatory scores, as well as inflammatory cell infiltration, especially for eosinophil infiltration. Chemokines, chemokine receptors and cytokines play important roles during eosinophil infiltration into the blood circulatory inflammatory tissues (Sannohe et al., 2003; Bisset and Schmid-Grendelmeier, 2005). Eotaxin is a member of the chemokine family, which can be produced in endothelial cells, smooth muscle cells, epithelial cells, alveolar macrophages and eosinophils (Scheerens et al., 2002). In asthma, eotaxin production is increased in bronchial airway lumen and mucosa. Eotaxin can positively participate in the pathogenesis of asthma through activating the eosinophil recruitment (Erin et al., 2002; Zietkowski et al., 2011). In this study, the eotaxin level is markedly up-regulated after OVA stimulation, while paeoniflorin treatment can suppress eotaxin production.

Additionally, IgE is also involved in the activation of eosinophils. Eosinophils can express surface membrane receptors showing high affinity and specificity to IgE. The interaction between the surface membrane receptors and antigen-binding IgE can induce the release of histamine, prostaglandin, leukotrienes and cytokines. These cytokines can activate the chemotaxis and phagocytosis of eosinophils and macrophages. Finally, the cytokine-induced reaction will lead to tissue inflammation (Bush, 2002; Stokes and Casale, 2015; Cui and Yin, 2018; Matucci et al., 2018). Some data indicates that, compared with the control group, the IgE level in OVA-exposed mice is markedly increased. However, the IgE level in the paeoniflorin treatment group at the dose of 50 mg/kg is distinctly reduced. In the pathogenesis of asthma, the Th2 and Th17 lymphocytes in the adaptive immune system can produce cytokines (such as IL-5, IL-13, and IL-17) to control disease (Ingram and Kraft, 2012; Russell and Brightling, 2016). In this study, compared with the control mice, the levels of these cytokines in OVA-induced mice are markedly increased. Compared with the model group, the levels of these cytokines are dramatically reduced.

To further discover the potential mechanism of action of paeoniflorin on asthma, we have adopted the transcriptomic and metabolomic techniques. In this study, we first described the metabolomic maps of lung tissues from model mice and paeoniflorin-treated mice, which demonstrated that the regulated metabolites mainly participate in fatty acid metabolism. Subsequently, we described the transcriptomic features of lung tissues, and discovered that the regulated transcripts are attributed to multiple functions, such as inflammatory response and adhesion plaque. Finally, we attempted to obtain the transcriptomic and metabolomic data for comprehensive consideration. The potential targets from both data sets were integrated, and the systemic results indicate that paeoniflorin may realize its improving effect on asthma depending on its systemic effect on fatty acid metabolism, inflammatory response and cell influence mechanism.

## Author Contributions

QShou, HF, and GC conceived and designed the experiments. QShou, LJ, JL, QShan, and QL performed the experiments. QShou and CC analyzed the data. QShou and HF contributed to reagents, materials, and analysis tools. HF and GC wrote the manuscript.

## Conflict of Interest Statement

The authors declare that the research was conducted in the absence of any commercial or financial relationships that could be construed as a potential conflict of interest.

## References

[B1] Bindea G. Mlecnik B. Hackl H. Charoentong P. Tosolini M. Kirilovsky A. (2009). ClueGO: a cytoscape plug-in to decipher functionally grouped gene ontology and pathway annotation networks. *Bioinformatics* 25 1091–1093. 10.1093/bioinformatics/btp101 19237447 PMC2666812

[B2] Bisset L. R. Schmid-Grendelmeier P. (2005). Chemokines and their receptors in the pathogenesis of allergic asthma: progress and perspective. *Curr. Opin. Pulm. Med.* 11 35–42. 10.1097/01.mcp.0000144502.50149.e0 15591886

[B3] Bush R. K. (2002). The use of anti-IgE in the treatment of allergic asthma. *Med. Clin. North Am.* 86 1113–1129. 10.1016/S0025-7125(02)00036-6 12428547

[B4] Chen J. Zhang M. Zhu M. Gu J. Song J. Cui L. (2018). Paeoniflorin prevents endoplasmic reticulum stress-associated inflammation in lipopolysaccharide-stimulated human umbilical vein endothelial cells via the IRE1alpha/NF-kappaB signaling pathway. *Food Funct.* 9 2386–2397. 10.1039/c7fo01406f 29594285

[B5] Cui L. Yin J. (2018). Association of serum specific IgE levels with asthma in autumn pollen-induced allergic rhinitis: a retrospective analysis. *J. Asthma* 18 1–7. 10.1080/02770903.2018.1466316 29667465

[B6] Erin E. M. Williams T. J. Barnes P. J. Hansel T. T. (2002). Eotaxin receptor (CCR3) antagonism in asthma and allergic disease. current drug targets. *Inflam. Allergy* 1 201–214. 10.2174/1568010023344715 14561201

[B7] Fang S. P. Tanaka T. Tago F. Okamoto T. Kojima S. (2005). Immunomodulatory effects of gyokuheifusan on INF-gamma/IL-4 (Th1/Th2) balance in ovalbumin (OVA)-induced asthma model mice. *Biol. Pharm. Bull.* 28 829–833. 10.1248/bpb.28.829 15863887

[B8] Gao J. Tarcea V. G. Karnovsky A. Mirel B. R. Weymouth T. E. Beecher C. W. (2010). Metscape: a cytoscape plug-in for visualizing and interpreting metabolomic data in the context of human metabolic networks. *Bioinformatics* 26 971–973. 10.1093/bioinformatics/btq048 20139469 PMC2844990

[B9] Gibeon D. Menzies-Gow A. (2013). Recent changes in the drug treatment of allergic asthma. *Clin. Med.* 13 477–481. 10.7861/clinmedicine.13-5-477 PMC4953799 24115705

[B10] He D. Y. Dai S. M. (2011). Anti-inflammatory and immunomodulatory effects of paeonia lactiflora pall., a traditional chinese herbal medicine. *Front. Pharmacol.* 2:10 10.3389/fphar.2011.00010 PMC3108611 21687505

[B11] Ingram J. L. Kraft M. (2012). IL-13 in asthma and allergic disease: asthma phenotypes and targeted therapies. *J. Allergy Clin. Immunol.* 130 829–842. 10.1016/j.jaci.2012.06.034 22951057

[B12] Lee B. Shin Y. W. Bae E. A. Han S. J. Kim J. S. Kang S. S. (2008). Antiallergic effect of the root of Paeonia lactiflora and its constituents paeoniflorin and paeonol. *Arch. Pharm. Res.* 31 445–450. 10.1007/s12272-001-1177-6 18449501

[B13] Li X. M. Zhang T. F. Sampson H. Zou Z. M. Beyer K. Wen M. C. (2004). The potential use of Chinese herbal medicines in treating allergic asthma. *Ann. Allergy Asthma Immunol.* 93 S35–S44. 10.1016/S1081-1206(10)61485-8 15330010

[B14] Liu Y. L. Zhang L. D. Ma T. M. Song S. T. Liu H. T. Wang X. (2018). Feishu acupuncture inhibits acetylcholine synthesis and restores muscarinic acetylcholine receptor m2 expression in the lung when treating allergic asthma. *Inflammation* 41 741–750. 10.1007/s10753-017-0726-y 29520557

[B15] Lu Y. Cai S. Nie J. Li Y. Shi G. Hao J. (2016). The natural compound nujiangexanthone A suppresses mast cell activation and allergic asthma. *Biochem. Pharmacol.* 100 61–72. 10.1016/j.bcp.2015.11.004 26571438

[B16] Luo W. Pan G. Huang H. Zheng P. Wei N. Zhang Y. (2017). A component-resolved diagnostic approach for a study on grass pollen allergens in chinese southerners with allergic rhinitis and/or asthma. *J. Vis. Exp.* 124:e55723 10.3791/55723 PMC5608237 28605372

[B17] Mao D. Tang R. Wu R. Hu H. Sun L. J. Zhu H. (2017). Prevalence trends in the characteristics of patients with allergic asthma in Beijing, 1994 to 2014. *Medicine (Baltimore)* 96:e7077 10.1097/md.0000000000007077 PMC5459741 28562576

[B18] Matucci A. Vultaggio A. Maggi E. Kasujee I. (2018). Is IgE or eosinophils the key player in allergic asthma pathogenesis? Are we asking the right question? *Respir. Res.* 19:113 10.1186/s12931-018-0813-0 PMC5992661 29879991

[B19] Morjaria J. B. Caruso M. Emma R. Russo C. Polosa R. (2018). Treatment of allergic rhinitis as a strategy for preventing asthma. *Curr. Allergy Asthma Rep.* 18:23 10.1007/s11882-018-0781-y 29574527

[B20] Radermecker C. Louis R. Bureau F. Marichal T. (2018). Role of neutrophils in allergic asthma. *Curr. Opin. Immunol.* 54 28–34. 10.1016/j.coi.2018.05.006 29883877

[B21] Ren S. Peng Z. Mao J. H. Yu Y. Yin C. Gao X. (2012). RNA-seq analysis of prostate cancer in the Chinese population identifies recurrent gene fusions, cancer-associated long noncoding RNAs and aberrant alternative splicings. *Cell Res.* 22 806–821. 10.1038/cr.2012.30 22349460 PMC3343650

[B22] Rosi E. Stendardi L. Binazzi B. Scano G. (2006). Perception of airway obstruction and airway inflammation in asthma: a review. *Lung* 184 251–258. 10.1007/s00408-005-2590-z 17235724

[B23] Russell R. Brightling C. E. (2016). Anti-IL-5 for severe asthma: aiming high to achieve success. *Chest* 150 766–768. 10.1016/j.chest.2016.06.013 27719806

[B24] Sannohe S. Adachi T. Hamada K. Honda K. Yamada Y. Saito N. (2003). Upregulated response to chemokines in oxidative metabolism of eosinophils in asthma and allergic rhinitis. *Eur. Respir. J.* 21 925–931. 10.1183/09031936.03.00028103a 12797483

[B25] Scheerens J. van Gessel S. B. Nijkamp F. P. Folkerts G. (2002). Eotaxin protein levels and airway pathology in a mouse model for allergic asthma. *Eur. J. Pharmacol.* 453 111–117. 10.1016/S0014-2999(02)02364-6 12393066

[B26] Serra D. S. Gomes M. D. M. Cavalcante F. S. A. Leal-Cardoso J. H. (2018). Essential oil of *Croton zehntneri* attenuates lung injury in the OVA-induced asthma model. *J. Asthma* 1–10. 10.1080/02770903.2018.1430828 [Epub ahead of print]. 29437496

[B27] Stokes J. R. Casale T. B. (2015). The use of anti-IgE therapy beyond allergic asthma. *J. Allergy Clin. Immunol. Pract.* 3 162–166. 10.1016/j.jaip.2014.10.010 25609342

[B28] Suarez-Farinas M. Lowes M. A. Zaba L. C. Krueger J. G. (2010). Evaluation of the psoriasis transcriptome across different studies by gene set enrichment analysis (GSEA). *PLoS One* 5:e10247 10.1371/journal.pone.0010247 PMC2857878 20422035

[B29] Tan H. T. Sugita K. Akdis C. A. (2016). Novel Biologicals for the treatment of allergic diseases and asthma. *Curr. Allergy Asthma Rep.* 16:70 10.1007/s11882-016-0650-5 27613653

[B30] Telia A. A. Telia A. Z. Machavariani K. Telia Z. (2018). Sublingual immunotherapy for allergic asthma and rhinitis. *Georgian Med. News* 276 123–130. 29697395

[B31] Wang Y. Zhu H. Tong J. Li Z. (2016). Ligustrazine improves blood circulation by suppressing platelet activation in a rat model of allergic asthma. *Environ. Toxicol. Pharmacol.* 45 334–339. 10.1016/j.etap.2016.06.016 27362664

[B32] Yan Y. Bao H. P. Li C. L. Shi Q. Kong Y. H. Yao T. (2018). Wentong decoction cures allergic bronchial asthma by regulating the apoptosis imbalance of EOS. *Chin. Med.* 13:21 10.1186/s13020-018-0180-2 PMC5907368 29713367

[B33] Yu M. Jia H. M. Cui F. X. Yang Y. Zhao Y. Yang M. H. (2017). The effect of chinese herbal medicine formula mkg on allergic asthma by regulating lung and plasma metabolic alternations. *Int. J. Mol. Sci.* 18:602 10.3390/ijms18030602 PMC5372618 28287417

[B34] Zhang T. Zhu Q. Shao Y. Wang K. Wu Y. (2017). Paeoniflorin prevents TLR2/4-mediated inflammation in type 2 diabetic nephropathy. *Biosci. Trends* 11 308–318. 10.5582/bst.2017.01104 28626209

[B35] Zietkowski Z. Skiepko R. Tomasiak-Lozowska M. M. Bodzenta-Lukaszyk A. (2011). Airway inflammation and eotaxin in exhaled breath condensate of patients with severe persistent allergic asthma during omalizumab therapy. *Adv. Med. Sci.* 56 318–322. 10.2478/v10039-011-0024-0 21940268

