# Supplementary material for: Integration of Metabolomics and Transcriptomics Reveals the Therapeutic Mechanism Underlying Paeoniflorin for the Treatment of Allergic Asthma
Source: Front Pharmacol. 2019 Jan 18;9:1531. doi: 10.3389/fphar.2018.01531 (PMC6362974; doi:10.3389/fphar.2018.01531)
Supplement: Supplementary file 2 [file Table_1.docx]

**Table S1. Identified metabolites, fold changes (FC) and *p*-values among control group, model group and paeoniflorin group in the positive ion mode**

|  |  |  | model vs control | | | paeoniflorin vs model | | | paeoniflorin vs control | | |
| --- | --- | --- | --- | --- | --- | --- | --- | --- | --- | --- | --- |
| No. | metabolites | VIP^a^ | FC^b^ | *p*-value^c^ | FDR^d^ | FC^b^ | *p*-value^c^ | FDR^d^ | FC^b^ | *p*-value^c^ | FDR^d^ |
| 1 | Phenol | 1.83 | 2.65 | 1.07E-05 | 5.06E-05 | 0.92 | 4.48E-01 | 6.08E-01 | 2.44 | 5.25E-05 | 2.50E-04 |
| 2 | Proline | 1.23 | 2.15 | 2.16E-02 | 2.56E-02 | 0.85 | 5.80E-01 | 7.35E-01 | 1.83 | 5.31E-02 | 6.31E-02 |
| 3 | Valine | 1.66 | 1.91 | 3.55E-04 | 6.74E-04 | 0.53 | 3.06E-04 | 5.81E-03 | 1.00 | 9.80E-01 | 9.80E-01 |
| 4 | Ornithine | 1.86 | 6.70 | 3.92E-06 | 2.48E-05 | 1.20 | 3.51E-01 | 5.13E-01 | 8.02 | 5.41E-05 | 2.06E-04 |
| 5 | 4-Aminobenzoic acid | 1.72 | 1.61 | 1.36E-04 | 4.30E-04 | 1.01 | 9.39E-01 | 9.91E-01 | 1.62 | 4.59E-03 | 7.27E-03 |
| 6 | spermidine | 1.91 | 1.74 | 4.95E-07 | 9.41E-06 | 1.13 | 1.99E-01 | 4.20E-01 | 1.96 | 5.52E-05 | 1.75E-04 |
| 7 | Histidine | 1.54 | 1.56 | 1.58E-03 | 2.51E-03 | 1.04 | 6.78E-01 | 8.05E-01 | 1.61 | 4.31E-05 | 2.73E-04 |
| 8 | Phenylalanine | 1.68 | 0.58 | 2.60E-04 | 7.06E-04 | 1.17 | 1.78E-01 | 4.23E-01 | 0.68 | 2.16E-04 | 5.14E-04 |
| 9 | Vitamin C | 1.61 | 23.9 | 6.61E-04 | 1.14E-03 | 0.42 | 2.45E-02 | 1.55E-01 | 10.11 | 4.58E-04 | 9.67E-04 |
| 10 | Tyrosine | 1.95 | 0.58 | 2.60E-04 | 6.18E-04 | 1.15 | 2.10E-04 | 3.99E-03 | 0.67 | 1.24E-01 | 3.37E-01 |
| 11 | Vitamin A | 1.34 | 2.17 | 9.98E-03 | 1.46E-02 | 0.71 | 1.30E-01 | 4.12E-01 | 1.53 | 9.41E-03 | 1.28E-02 |
| 12 | 13-KODE | 1.78 | 2.75 | 3.68E-05 | 1.40E-04 | 1.80 | 3.83E-02 | 1.82E-01 | 4.95 | 1.09E-03 | 2.07E-03 |
| 13 | γ-linolenic acid | 1.67 | 2.39 | 2.93E-04 | 6.18E-04 | 1.27 | 1.30E-01 | 3.54E-01 | 3.04 | 3.11E-05 | 2.95E-04 |
| 14 | Reduced Glutathione | 1.09 | 1.27 | 4.87E-02 | 5.44E-02 | 0.87 | 2.57E-01 | 4.44E-01 | 1.11 | 2.50E-01 | 2.64E-01 |
| 15 | Guanosine monophosphate | 1.34 | 0.45 | 1.02E-02 | 1.38E-02 | 1.01 | 9.64E-01 | 9.64E-01 | 0.45 | 1.41E-02 | 1.79E-02 |

^a^ VIP was obtained from PLS-DA; ^b^ FC was calculated based on mean ratios for model vs control, paeoniflorin vs model or paeoniflorin vs control. FC with a value greater than 1.0 indicates a higher intensity between model vs control, between paeoniflorin vs model or between paeoniflorin vs control, while a FC value less than 1.0 indicates a lower intensity of the lipid species between model vs control, between paeoniflorin vs model or between paeoniflorin vs control; ^c^ *p*-values are calculated from a one-way anova; ^d^FDR value was obtained from the adjusted *p* value using Benjamini Hochberg method.
