# Supplementary material for: Integration of Metabolomics and Transcriptomics Reveals the Therapeutic Mechanism Underlying Paeoniflorin for the Treatment of Allergic Asthma
Source: Front Pharmacol. 2019 Jan 18;9:1531. doi: 10.3389/fphar.2018.01531 (PMC6362974; doi:10.3389/fphar.2018.01531)
Supplement: Supplementary file 3 [file Table_2.docx]

**Table S2. Identified metabolites, fold changes (FC) and *p*-values among control group, model group and paeoniflorin group in the negative ion mode**

|  |  |  | model vs Control | | | paeoniflorin vs model | | | paeoniflorin vs control | | |
| --- | --- | --- | --- | --- | --- | --- | --- | --- | --- | --- | --- |
| No. | metabolites | VIP^a^ | FC^b^ | *p*-value^c^ | FDR^d^ | FC^b^ | *p*-value^c^ | FDR^d^ | FC^b^ | *p*-value^c^ | FDR^d^ |
| 1 | Prostaglandin E1 | 1.40 | 1.41 | 5.19E-04 | 9.86E-04 | 1.24 | 2.79E-02 | 6.62E-02 | 1.74 | 4.48E-05 | 1.42E-04 |
| 2 | Malic Acid | 1.11 | 0.64 | 1.40E-02 | 1.27E-02 | 1.22 | 3.60E-01 | 3.26E-01 | 0.78 | 1.94E-01 | 1.60E-01 |
| 3 | (10E,12Z)-9-Hydroperoxy-10,12-octadecadienoic acid | 1.40 | 2.34 | 4.91E-04 | 1.04E-03 | 2.03 | 4.66E-02 | 8.05E-02 | 4.76 | 4.14E-03 | 4.91E-03 |
| 4 | 9,12,15-Octadecatrienoic acid | 1.41 | 1.43 | 4.23E-04 | 1.15E-03 | 1.44 | 6.16E-03 | 4.85E-02 | 2.06 | 7.14E-05 | 1.94E-04 |
| 5 | 3-Oxalomalic acid | 1.23 | 0.48 | 4.63E-03 | 5.50E-03 | 1.44 | 3.46E-01 | 3.29E-01 | 0.69 | 9.79E-02 | 8.45E-02 |
| 6 | 5,6-Dihydroretinoic acid | 1.59 | 2.17 | 3.62E-06 | 3.44E-05 | 1.20 | 1.91E-02 | 7.25E-02 | 2.61 | 8.77E-11 | 1.67E-09 |
| 7 | 9E,12E-octadecadienoic acid | 1.46 | 0.73 | 1.73E-04 | 5.49E-04 | 0.98 | 7.31E-01 | 5.56E-01 | 0.71 | 3.65E-04 | 7.72E-04 |
| 8 | Glutamine | 1.48 | 0.74 | 1.18E-04 | 4.50E-04 | 1.30 | 2.09E-02 | 4.62E-02 | 0.96 | 6.80E-01 | 5.17E-01 |
| 9 | Lactic Acid | 1.30 | 0.33 | 2.17E-03 | 2.94E-03 | 1.16 | 8.05E-01 | 5.46E-01 | 0.38 | 1.76E-02 | 1.67E-02 |
| 10 | Tryptophan | 1.03 | 1.53 | 2.48E-02 | 2.05E-02 | 1.33 | 1.15E-01 | 1.45E-01 | 2.03 | 5.02E-04 | 9.54E-04 |
| 11 | Gamma-Hydroxybutyric acid | 1.28 | 0.48 | 2.75E-03 | 3.48E-03 | 1.19 | 1.85E-01 | 1.95E-01 | 0.57 | 9.64E-03 | 1.08E-02 |
| 12 | Hypoxanthin | 1.36 | 0.78 | 9.84E-04 | 1.44E-03 | 1.28 | 6.29E-02 | 9.96E-02 | 1.00 | 9.65E-01 | 6.55E-01 |
| 13 | Threonine | 1.41 | 1.64 | 4.29E-04 | 1.02E-03 | 1.04 | 7.52E-01 | 5.49E-01 | 1.71 | 2.91E-03 | 4.26E-03 |
| 14 | Leucine | 1.57 | 2.63 | 9.45E-06 | 5.98E-05 | 0.92 | 7.07E-01 | 5.60E-01 | 2.43 | 1.39E-02 | 1.47E-02 |
| 15 | Leukotriene B4 | 1.71 | 2.76 | 1.62E-10 | 3.08E-09 | 1.18 | 9.39E-03 | 4.95E-02 | 3.25 | 1.78E-09 | 1.69E-08 |
| 16 | Pyroglutamic acid | 1.08 | 0.80 | 1.71E-02 | 1.48E-02 | 1.03 | 7.93E-01 | 5.58E-01 | 0.82 | 3.31E-02 | 3.00E-02 |
| 17 | N-Acetyl-L-aspartic acid | 1.49 | 1.77 | 8.35E-05 | 3.97E-04 | 0.93 | 4.81E-01 | 3.97E-01 | 1.65 | 3.67E-03 | 4.98E-03 |
| 18 | Palmitic acid | 1.11 | 1.22 | 1.37E-02 | 1.30E-02 | 1.08 | 2.98E-01 | 2.98E-01 | 1.32 | 1.65E-03 | 2.61E-03 |
| 19 | Prostaglandin G2 | 1.21 | 1.33 | 5.77E-03 | 6.44E-03 | 1.17 | 6.76E-02 | 9.88E-02 | 1.55 | 4.02E-05 | 1.53E-04 |
| 20 | Pyruvic acid | 1.37 | 3.09 | 7.81E-04 | 1.35E-03 | 0.76 | 1.38E-01 | 1.55E-01 | 2.33 | 7.75E-06 | 4.91E-05 |
| 21 | Stearic acid | 1.19 | 1.27 | 6.71E-03 | 7.08E-03 | 0.94 | 4.09E-01 | 3.53E-01 | 1.20 | 3.75E-03 | 4.76E-03 |
| 22 | Glutamic acid | 1.99 | 0.85 | 3.33E-02 | 2.64E-02 | 1.19 | 4.61E-02 | 4.76E-02 | 1.01 | 9.26E-01 | 6.51E-01 |

^a^ VIP was obtained from PLS-DA; ^b^ FC was calculated based on mean ratios for model vs control, paeoniflorin vs model or paeoniflorin vs control. FC with a value greater than 1.0 indicates a higher intensity between model vs control, between paeoniflorin vs model or between paeoniflorin vs control, while a FC value less than 1.0 indicates a lower intensity of the lipid species between model vs control, between paeoniflorin vs model or between paeoniflorin vs control; ^c^ *p*-values are calculated from a one-way anova; ^d^ FDR value was obtained from the adjusted *p* value using Benjamini Hochberg method.
