# Supplementary material for: Integration of Metabolomics and Transcriptomics Reveals the Therapeutic Mechanism Underlying Paeoniflorin for the Treatment of Allergic Asthma
Source: Front Pharmacol. 2019 Jan 18;9:1531. doi: 10.3389/fphar.2018.01531 (PMC6362974; doi:10.3389/fphar.2018.01531)
Supplement: Supplementary file 4 [file Table_3.docx]

**Table S3 The changed pathways in the model and paeoniflorin groups at the metabolomic level**

| **Total** | **Hits** | **Expect** | **P value** | **Holm P** | **FDR** | **Details** |
| --- | --- | --- | --- | --- | --- | --- |
| AMMONIA RECYCLING | 18 | 4 | 0.7 | 0.00393 | 0.311 | 0.116 |
| GLUTAMATE METABOLISM | 18 | 4 | 0.7 | 0.00393 | 0.311 | 0.116 |
| GLUTATHIONE METABOLISM | 10 | 3 | 0.389 | 0.00533 | 0.41 | 0.116 |
| UREA CYCLE | 20 | 4 | 0.778 | 0.0059 | 0.448 | 0.116 |
| ALANINE METABOLISM | 6 | 2 | 0.233 | 0.0199 | 1 | 0.315 |
| CYSTEINE METABOLISM | 8 | 2 | 0.311 | 0.0354 | 1 | 0.383 |
| MALATE-ASPARTATE SHUTTLE | 8 | 2 | 0.311 | 0.0354 | 1 | 0.383 |
| PYRUVATE METABOLISM | 20 | 3 | 0.778 | 0.0388 | 1 | 0.383 |
| HISTIDINE METABOLISM | 11 | 2 | 0.428 | 0.0647 | 1 | 0.532 |
| ARGININE AND PROLINE METABOLISM | 26 | 3 | 1.01 | 0.0756 | 1 | 0.532 |
| GLUCOSE-ALANINE CYCLE | 12 | 2 | 0.467 | 0.0758 | 1 | 0.532 |
| GLUCONEOGENESIS | 27 | 3 | 1.05 | 0.0828 | 1 | 0.532 |
| PHENYLALANINE AND TYROSINE METABOLISM | 13 | 2 | 0.505 | 0.0875 | 1 | 0.532 |
| INSULIN SIGNALLING | 19 | 2 | 0.739 | 0.166 | 1 | 0.938 |
| CATECHOLAMINE BIOSYNTHESIS | 5 | 1 | 0.194 | 0.18 | 1 | 0.949 |
| CITRIC ACID CYCLE | 23 | 2 | 0.894 | 0.224 | 1 | 1 |
| GLYCINE, SERINE AND THREONINE METABOLISM | 26 | 2 | 1.01 | 0.268 | 1 | 1 |
| ALPHA LINOLENIC ACID AND LINOLEIC ACID METABOLISM | 9 | 1 | 0.35 | 0.301 | 1 | 1 |
| RNA TRANSCRIPTION | 9 | 1 | 0.35 | 0.301 | 1 | 1 |
| ASPARTATE METABOLISM | 12 | 1 | 0.467 | 0.381 | 1 | 1 |
| GLYCEROLIPID METABOLISM | 13 | 1 | 0.505 | 0.405 | 1 | 1 |
| VALINE, LEUCINE AND ISOLEUCINE DEGRADATION | 36 | 2 | 1.4 | 0.414 | 1 | 1 |
| ARACHIDONIC ACID METABOLISM | 37 | 2 | 1.44 | 0.428 | 1 | 1 |
| PROPANOATE METABOLISM | 18 | 1 | 0.7 | 0.514 | 1 | 1 |
| RETINOL METABOLISM | 18 | 1 | 0.7 | 0.514 | 1 | 1 |
| PURINE METABOLISM | 45 | 2 | 1.75 | 0.533 | 1 | 1 |
| GLYCOLYSIS | 21 | 1 | 0.817 | 0.57 | 1 | 1 |
| METHIONINE METABOLISM | 24 | 1 | 0.933 | 0.619 | 1 | 1 |
| FATTY ACID ELONGATION IN MITOCHONDRIA | 26 | 1 | 1.01 | 0.649 | 1 | 1 |
| FATTY ACID METABOLISM | 29 | 1 | 1.13 | 0.69 | 1 | 1 |
| TRYPTOPHAN METABOLISM | 34 | 1 | 1.32 | 0.748 | 1 | 1 |
| PYRIMIDINE METABOLISM | 36 | 1 | 1.4 | 0.768 | 1 | 1 |
| TYROSINE METABOLISM | 38 | 1 | 1.48 | 0.786 | 1 | 1 |
